# Genomic and transcriptomic landscapes of Epstein-Barr virus in extranodal natural killer T-cell lymphoma

**DOI:** 10.1038/s41375-018-0324-5

**Published:** 2018-12-13

**Authors:** Rou-Jun Peng, Bo-Wei Han, Qing-Qing Cai, Xiao-Yu Zuo, Tao Xia, Jie-Rong Chen, Li-Na Feng, Jing Quan Lim, Shu-Wei Chen, Mu-Sheng Zeng, Yun-Miao Guo, Bo Li, Xiao-Jun Xia, Yi Xia, Yurike Laurensia, Burton Kuan Hui Chia, Hui-Qiang Huang, Ken He Young, Soon Thye Lim, Choon Kiat Ong, Yi-Xin Zeng, Jin-Xin Bei

**Affiliations:** 1 Sun Yat-Sen University Cancer Center, State Key Laboratory of Oncology in South China, Collaborative Innovation Center for Cancer Medicine, Guangzhou, China; 2 Department of Oncology, Affiliated Hospital of Jining Medical University, Jining, Shandong China; 3 Division of Cellular and Molecular Research, National Cancer Centre Singapore, Singapore, Singapore; 4 Department of Biochemistry and Molecular Biology, Zhongshan School of Medicine, Sun Yat-Sen University, Guangzhou, 510080 China; 5 RNA Biomedical Institute, Sun Yat-Sen Memorial Hospital, Sun Yat-Sen University, Guangzhou, 510120 China; 6 Department of Hematopathology, The University of Texas MD Anderson Cancer Center, Houston, TX USA; 7 Division of Medical Oncology, National Cancer Center Singapore, Singapore, Singapore; 8 SingHealth Duke-NUS Blood Cancer Centre, Singapore, Singapore; 9 Duke-NUS Medical School, Singapore, Singapore; 10 Genome Institute of Singapore, A*STAR, Singapore, Singapore; 11 Center for Precision Medicine, Sun Yat-Sen University, Guangzhou, China

**Keywords:** Cancer genomics, Tumour virus infections

## Abstract

Extranodal natural killer T-cell lymphoma (nasal type; NKTCL) is an aggressive malignancy strongly associated with Epstein-Barr virus (EBV) infection. However, the role of EBV in NKTCL development is unclear, largely due to the lack of information about EBV genome and transcriptome in NKTCL. Here, using high-throughput sequencing, we obtained whole genome (*n* = 27) and transcriptome datasets (*n* = 18) of EBV derived from NKTCL tumor biopsies. We assembled 27 EBV genomes and detected an average of 1,152 single nucleotide variants and 44.8 indels (<50 bp) of EBV per sample. We also identified frequent focal EBV genome deletions and integrated EBV fragments in the host genome. Moreover, Phylogenetic analysis revealed that NKTCL-derived EBVs are closely clustered; transcriptome analysis revealed less activation of both latent and lytic genes and larger amount of T-cell epitope alterations in NKTCL, as compared with other EBV-associated cancers. Furthermore, we observed transcriptional defects of the BARTs miRNA by deletion, and the disruption of host *NHEJ1* by integrated EBV fragment, implying novel pathogenic mechanisms of EBV. Taken together, we reported for the first time global mutational and transcriptional profiles of EBV in NKTCL clinical samples, revealing important somatic events of EBV and providing insights to better understanding of EBV’s contribution in tumorigenesis.

## Introduction

Extranodal natural killer T-cell lymphoma (NKTCL, nasal type) is an aggressive form of lymphoma with geographical prevalence in Asian and South American populations [1–3]. Multiple factors have been implicated in the development of NKTCL. Our recent genome-wide association study identified *HLA-DPB1* as a susceptibility gene predisposing individuals to NKTCL [4]. Acquired somatic alterations, including *DDX3X*, *TP53*, and *STAT3* mutations have been determined in NKTCL tumors and the related signaling pathways, such as JAK-STAT, NF-κB, and MAPK pathways have been implicated in its pathogenesis [5, 6]. Epstein-Barr virus (EBV) has been strongly associated with NKTCL, mostly based on the observation of EBV molecules in the tumor tissue, and the correlations between EBV load and disease diagnosis as well as prognosis [1, 2, 7]. In addition, environmental factors such as exposures to chemical solvents increased risk of NKTCL [3].

Apart from NKTCL, EBV infection has been linked with various malignancies, including Burkitt lymphoma (BL), nasopharyngeal carcinoma (NPC), and gastric carcinoma (GC) [8, 9]. On the other hand, EBV is a ubiquitous gamma-herpesvirus infecting more than 95% of the worldwide population, whereas most of the EBV-related malignancies show remarkably high-incidence rates in the prevalent regions, respectively, making the underlying details of its association with cancers enigmatic [10]. Genomic diversity of EBV has been demonstrated among individuals with various types of cancers or with normal infection from different geographic regions [8, 11]. Moreover, recent study showed that recombinant EBV strains originated from different types of cancers exhibited different capabilities to infect epithelial cells and transform B cells [12]. These findings lead to a hypothesis that there might be disease-specific EBV strains conferring risks to the disease. Supportively, an EBV subtype based on *RPMS1* mutation has been specifically associated with NPC [13].

Little is known about the genomic and transcriptomic profiles of EBV in NKTCL. Sequence variations of *LMP1* have been demonstrated in NKTCL and its variant of 30-bp deletion is associated with poor prognosis of patients with NKTCL, which might serve as a potential marker to monitor treatment [14]. This is in line with the findings that LMP1 is a key latent protein with abilities to promote cell proliferation and inhibit cell apoptosis in NKTCL [15–17].

Here, by retrieving whole-genome DNA and RNA sequencing data derived from NKTCL tumor biopsy samples, we assembled 27 NKTCL-derived EBV genome sequences, and characterized genomic alterations, gene expression patterns, and T-cell epitope variations for NKTCL-derived EBV. We also compared mutational and expressional spectrum among three common EBV-related cancers in Asia, namely NKTCL, NPC, and GC. Together, we presented the genetic and transcriptomic landscape of EBV in NKTCL for the first time, providing insights into the pathogenic role of EBV in NKTCL.

## Materials and methods

### Patient recruitment and sample processing

A total of 27 patients were recruited from the Sun Yat-sen University Cancer Center (SYSUCC), China during October 01, 2015 to December 30, 2016, and National Cancer Center Singapore (NCCS) during January 01, 2005 to December 31, 2013, respectively. NKTCL was diagnosed according to the 2008 World Health Organization classification with cytotoxic, CD3ε+ and *EBER*+ phenotype, and clinical information were summarized in Table S1. The study was approved by the Institutional Review Boards from SYSUCC (YB2015-015-01) and SingHealth (2004/407/F). Tumor tissue biopsies prior to treatment were collected from the patients and written informed consent was obtained. The tumor purity was high (80–100%) in tested samples as reviewed independently by two pathologists. Tumor biopsies were soaked in RNAlater (Thermo Fisher) and fresh-frozen.

### Whole-genome sequencing and whole-transcriptome sequencing

Genomic DNA was extracted from fresh-frozen tissue using DNeasy Blood & Tissue Kit (QIAGEN) according to the manufacturer’s protocol, respectively. For whole-genome sequencing, 200 ng of genomic DNA was fragmented by sonication and was processed with library preparation following instructions by TruSeq Nano DNA LT Library Prep Kit (Illumina). The library was subjected for high-throughput sequencing with pair-end of 150 bp using the Hiseq X sequencer (Illumina).

For whole-transcriptome sequencing, total RNA was extracted from tumor specimens by using RNeasy Mini Kit (Qiagen), and 1 μg total RNA was subjected for the depletion of ribosomal RNAs by using Ribo-Zero Magnetic Kit (Illumina), followed by library preparation according to the manufacturer’s instructions as in the TruSeq RNA Library Prep Kit (Illumina). Subsequently, the library was loaded for high-throughput sequencing with pair-end of 150 bp using the Hiseq X sequencer (Illumina).

### Genome assembly and phylogenetic analysis of EBV sequences

After removal of sequencing reads with low quality, sequencing reads were aligned to the aggregated EBV reference sequences containing 164 EBV genomes retrieved from NCBI Genbank using BWA [18], and the aligned reads were assembled using SPAdes 3.9.1 [19]. Scaffolds were aligned to the EBV reference NC_007605.1 and quality assessment was carried out using Quast [20].

To generate phylogenetic tree of EBV, coding domain sequence (CDS) in non-repeat region was extracted from each EBV genome, and multiple alignment was performed using MAFFT v7 with default settings [21]. Phylogenetic trees were visualized by FastTree2 and ggtree [22].

### Mutations calling of EBV sequences

EMBOSS Stretcher was performed for pairwise alignment of each EBV sequence against the reference EBV sequence (NC_007605.1) and for the calculation of sequence similarity. Mutations including single nucleotide variants (SNVs), small insertions and deletions (<50 bp) (Indels) were called using in-house Python scripts. Changes of amino acid sequence were annotated using ANNOVAR [23]. To enrich likelihood of tumor-related mutations, variant would be excluded if the variant had high mutation frequencies (>5%) in the 38 non-cancer EBV isolates retrieved from NCBI GenBank. In addition, EBV mutations for samples, including NPC and GC were called with the same pipeline.

### Genomic deletion and host integration of EBV sequence

Read pairs of PCR duplicates were removed from further analyses. Genomic deletion of EBV was tagged if a read was identified as splicing junction, which cannot be mapped contiguously and completely to the reference EBV sequence; and the deletion size was calculated from the length of the genomic region spanning the splicing junction. To identify host-virus integration site, after removal of read pairs with both mapping entirely to either human or EBV, reads were aligned to human genome (hg19) and EBV genome (NC_007605.1) separately. A read or read pairs mapped to both human and EBV simultaneously was flagged; and the location and orientation of the integration were determined accordingly using in-house scripts. For all the alignments, only the regions with GC content between 20 and 80% were counted, and repeat regions were removed if annotated by RepeatMasker [24].

### Transcriptome analysis

Pair-end reads with high quality were aligned to ribosome RNAs using Bowtie 2 [25], and reads after removal of those being aligned as ribosome RNAs were realigned to the referenced EBV sequence (NC_007605.1) using HISAT2 with default settings [26]. HTseq was used to quantitate the read counts of each gene [27]. The expression levels of genes were normalized using Reads per Kilobase per Million mapped reads (RPKM), to minimize the potential effect of tumor purity. In addition, RNA sequencing data obtained via public database were processed with the same pipeline.

### T-cell epitope analysis

Known T-cell epitope sequences and genomic loci were annotated based on previous studies [28]. Briefly, by using SNVs and small Indels based on whole-genome sequencing data, amino acid sequence changes were annotated by ANNOVAR [23]. Mutations of T-cell epitopes were identified according to the changes of T-cell epitope sequences, and only genes with at least 10 RPKM in the NKTCL transcriptome data were considered.

### Statistical analysis

Student's t-test was used for comparing number of variants between two groups, and Dunnett's multiple comparison tests were used for comparing number of variants among three groups after statistically compared variance of each group. To compare the mutation frequencies and the T-cell epitope variation frequencies between two groups, odd ratio was calculated and Fisher's exact test was conducted using R. Expression correlation between two genes was measured by Pearson correlation coefficient.

### Accession numbers

All EBV sequences have been deposited in the NCBI GenBank (https://www.ncbi.nlm.nih.gov/Genbank) and individual sample accession numbers are listed in Table S2. The key raw data of this study have been uploaded onto the Research Data Deposit (RDD; http://www.researchdata.org.cn/) with an RDD number of RDDB2018000485.

### Code availability

In-house codes used to identify mutation, genomic deletion and host integration of EBV sequence as abovementioned have been uploaded to https://github.com/hanbw/ebv_script.

## Results

### De novo assembly of EBV genome in NKTCL tumor

To obtain full-length sequence of EBV in NKTCL, we retrieved whole-genome sequencing (WGS) data of 27 EBV-positive NKTCL tumor samples from Southern China (*n* = 15) and Singapore (*n* = 12), respectively. Clean reads of WGS sequences were aligned to human genome (hg19) and EBV reference genome (GenBank ID: NC _007605.1), respectively. The average percentage of EBV sequences in WGS data is 0.45% (0.03 ~ 1.06%), and the coverage depth is 222.2X in average (26.7X ~ 612.8X).

**Fig. 1:**
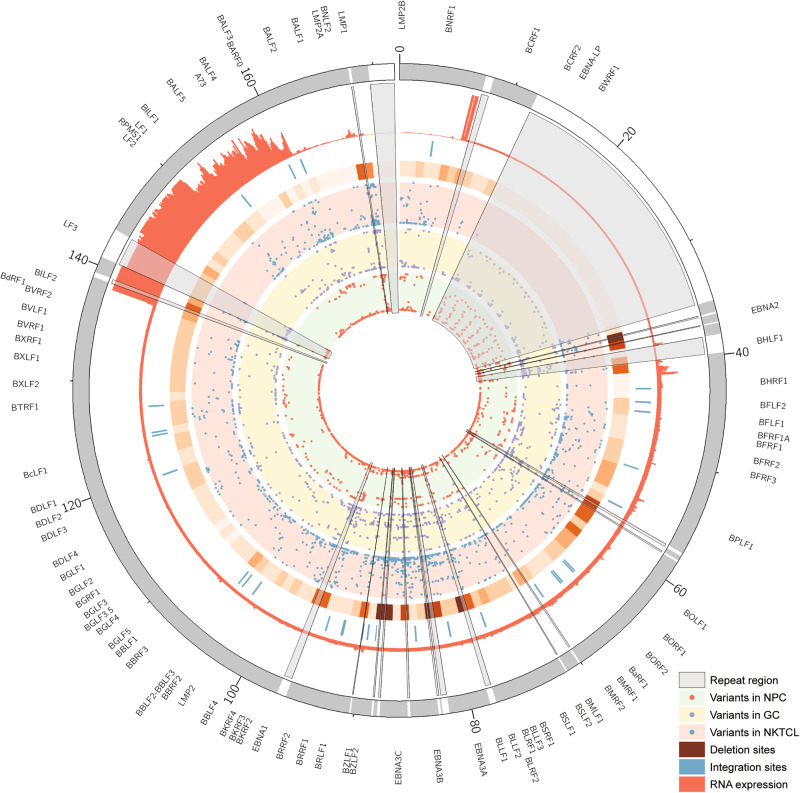
The landscape of EBV genomic mutations and transcription in NKTCL. The Circos plots showed tracks from inner to outer, representing mutational frequency of each EBV variant in NPC, GC, and NKTCL, frequency of deletion (higher with deeper color), EBV-host integration site, transcriptional coverage in NKTCL-derived EBV and EBV reference genome coordinate. Repeat regions of EBV genome were marked using gray sectors; and ticks outside indicates EBV genes

EBV sequences were assembled into several large contiguous DNA sequences (scaffolds), which were then aligned to the EBV reference genome. In average, the total aligned length for each genome is 142,924.7 bp (ranging from 126,660 bp to 148,128 bp), and the NGA50 is 59,527.4 bp (ranging from 3655 bp to 103,975 bp). As ~34 kb of 172 kb of EBV genome are repeat regions, which could not be properly assembled with short-reads sequencing technology, we assigned “N” for these regions and subsequently joined the scaffolds, resulting in EBV genomes with ~172 kb in length (Fig. 1).

### Single nucleotide variant (SNV) of EBV genome in NKTCL

**Fig. 2:**
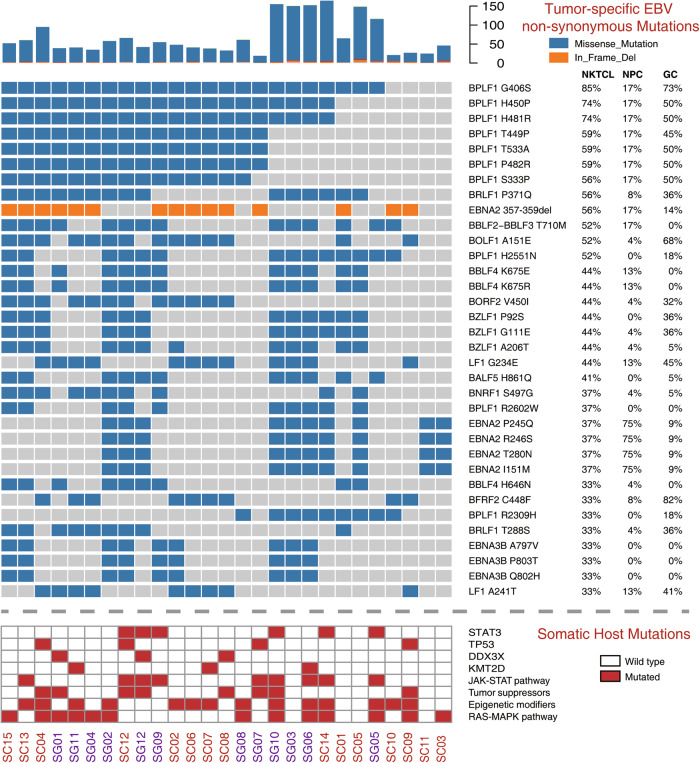
Non-synonymous EBV mutations in NKTCL. The histogram on top panel shows the number of total non-synonymous mutations in NKTCL-derived EBV, the mutational heatmap (top) indicates the most recurrent mutations NKTCL-derived EBV, and the frequencies of the mutation for NKTCL, NPC, and GC are listed on the right. The heatmap (bottom) indicates somatic host mutations in known driver genes and pathways (right) in NKTCL samples. Sample identifiers are listed at bottom

Among the 27 NKTCL samples, in average 1,152 EBV SNVs for each sample were determined by aligning the viral reads against the EBV reference genome (NC_007605.1). New mutation hotspots were observed at *BPLF1* and *BDLF2/3* regions, other than those at *EBNAs*, *LMP1*, and *LMP2* that were consistently reported in EBV derived from other cancers [8] (Figure S1). The most frequent tumor-specific non-synonymous mutations in NKTCL-derived EBV were located at *BPLF1* gene (position 49,790 bp ~ 59,239 bp; Figs. 1 and 2). No significant difference in the total numbers of point mutations (either all mutations or tumor-specific non-synonymous) of EBV was found between samples as grouped according to their collection points (Southern China versus Singapore; Figure S2A and B), gender (male versus female), age (below or above 40), and clinical stages (1 & 2 versus 3 & 4; Figure S2C), respectively. However, a variant of LMP1 (V43L) was significantly enriched in the Singapore samples (*p* = 0.028; Figure S2D) and no mutations associated with gender, age, and staging were identified (data not shown).

Somatic host mutation profiles of NKTCL patients were also analyzed. Similar to previously published studies [5, 29, 30], genes, including *STAT3*, *TP53*, *DDX3X*, and *KMT2D* were mutated in our NKTCL samples, and recurrent mutations were most frequently found in JAK-STAT pathway, tumor suppressors, epigenetic modifiers, and RAS-MAPK pathway (Figure S3). Although total mutation numbers of EBV showed no correlation with mutations of a particular gene or pathway (Fig. 2), patients with JAK-STAT pathway mutations showed higher mutation rates on a set of EBV genes, including *BBLF* family, *BYRF1*, and so on (Figure S4).

### Insertions and deletions of EBV genome in NKTCL

**Fig. 3:**
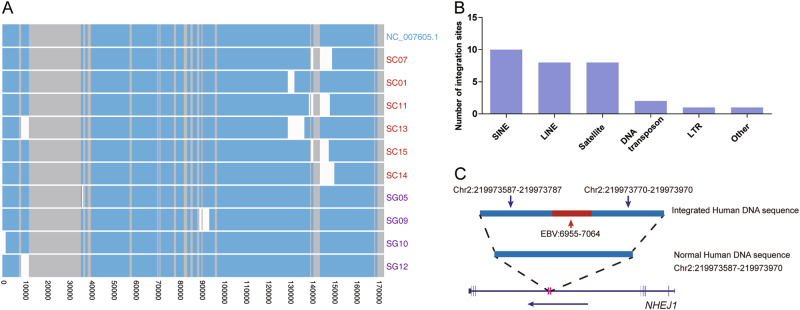
Deletions and host-integrations of EBV in NKTCL. **a** Whole-genome alignment of EBV clones with large deletions using NC_007605.1 as reference. Bars with blue, gray, and white colors indicate observed, repeat and deletion regions, respectively. Samples with multiple clones of impaired and intact EBV genome are presented separately (Figure S6). **b** Number of EBV-host integration sites as grouped by gene categories. **c** Schematic presentation of EBV-host integration site in human *NHEJ1* gene; coordinates are in human genome hg19

Insertions and deletions were detected in NKTCL-derived EBV genomes. An average of 44.8 small indels (<50 bp) of EBV were found in each NKTCL sample, and the 30-bp deletion of *LMP1* was commonly found in the samples (21/27), with a frequency consistent with the previous study revealed by using Sanger sequencing [31]. Frequent small deletions were also identified in the mutation hotspots, such as in positions 36 ~ 37 kb (*EBNA2*), 77 ~ 78 kb (*BLLF1/2*), 81 ~ 88 kb (*EBNA3s*), and 167 ~ 168 kb (*LMP1*) (Fig. 1 and S5). Large deletions of EBV (>1 kb) were found in 10 of 27 NKTCL samples, without any sequencing coverage in the deleted regions (Fig. 3a). Interestingly, the deletions were enriched in the locus 130 ~ 150 kb (6 out of 10 samples). Among them, four EBV genomes resulted in the partial loss of coding regions for BART miRNAs and introns of *RPMS1*; and the other two were lack of the upstream sequence of *RPMS1* (Fig. 3a). Moreover, co-existence of both long-fragment-deleted and full-length EBV genomes was identified in three samples, suggesting the presence of multiple EBV clones in the individual NKTCL tissues. Interestingly, the impaired EBV clones of the three samples were missing region spanning BART miRNAs and upstream of *RPMS1* (Figure S6).

### Host integrations of EBV sequence in NKTCL

We screened for potential integration sites between EBV and human genomes, with evidence of more than two chimeric sequence reads. A total of 31 EBV-host integration sites were identified in eight samples, which were enriched in the repeat regions of human genome, such as SINE, LINE, and satellite, etc. (Fig. 3b), with a similar scenario as described in a previous study on hepatitis B virus (HBV) [32]. Notably, an insertion of EBV fragment of 109 bp into the intron of human gene *NHEJ1* was identified with 13 supportive read pairs (Fig. 3c), and was further validated using Sanger Sequencing (Figure S7). No other chimeric pair-end reads were found tiling farer across the integration sites, meaning lack of evidence for the integration of longer EBV genome.

### Genome diversity of EBV derived from NKTCL and other EBV-associated diseases

**Fig. 4:**
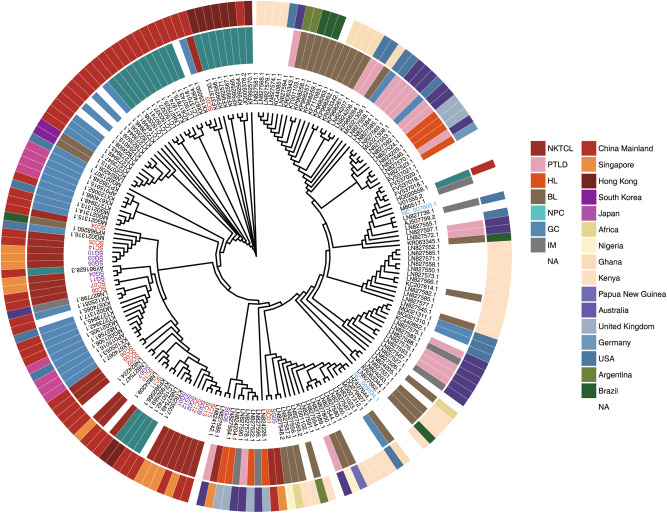
Phylogenetic analysis of EBV isolates from multiple geographic regions and different diseases. Coding sequences outside repeat regions of EBV genome were used for phylogenetic analysis. Heatmap with shorter bars at the outer ring indicate geographic origins for the EBV isolates and heatmap with longer bars at the inner ring indicate type of disease for the EBV isolates. The exact regions and types of the diseases are listed at the right panels. NKTCL natural killer T-cell lymphoma; PTLD posttransplant lymphoproliferative disorders; HL Hodgkin lymphoma; BL Burkitt lymphoma; NPC nasopharyngeal carcinoma; GC gastric carcinoma; IM infectious mononucleosis

To determine the sequence diversity of EBV, we compared the sequences between NKTCL-derived EBV and 164 EBV genome sequences from public database. In average, EBV derived from NKTCL shared lower sequence similarities to the referenced EBV sequence (average 97.4%, ranging from 94.2 to 98.9%), as compared with EBV derived from NPC (98.5, ranging from 96.8 to 99.2%) and GC (98.7%, ranging from 96.9 to 99.2%), respectively. Mutational hotspots are similar among NKTCL, NPC, and GC samples, except that higher mutation rates were observed at *EBNA3* and *BPLF1* loci in NKTCL-derived EBV and at *LMP2* locus in NPC and GC (Figure S1). Phylogenetic analysis revealed clear clustering of EBV isolates firstly according to their respective geographic origin; and moreover, EBV isolates derived from one disease tend to cluster together and deviate apart from those of other cancers, suggesting the existence of unique sequence features of EBV in each disease (Fig. 4).

### Disease-specific EBV mutations

The mutation profiles of EBV derived from NKTCL, NPC, and GC samples were compared. Significantly higher number of non-synonymous EBV mutations was observed in NKTCL than in NPC or GC samples (Figure S8). Thirty-three variants in 20 EBV genes were found frequently mutated in NTKCL than NPC (Figure S9); whereas, 14 mutations in 10 EBV genes were frequently found in NKTCL as compared with that of GC (*p* < 0.001; Figure S10). Notably, variants in *BBLF2/3* and *BPLF1* are significantly enriched in NKTCL, as compared with GC and NPC, respectively. Unsurprisingly, a cluster of disease-specific mutations were commonly observed in the *EBNAs* loci, as the *EBNAs* are known as one of the mutation hotspots.

### Expression profile of EBV genes in NKTCL

**Fig. 5:**
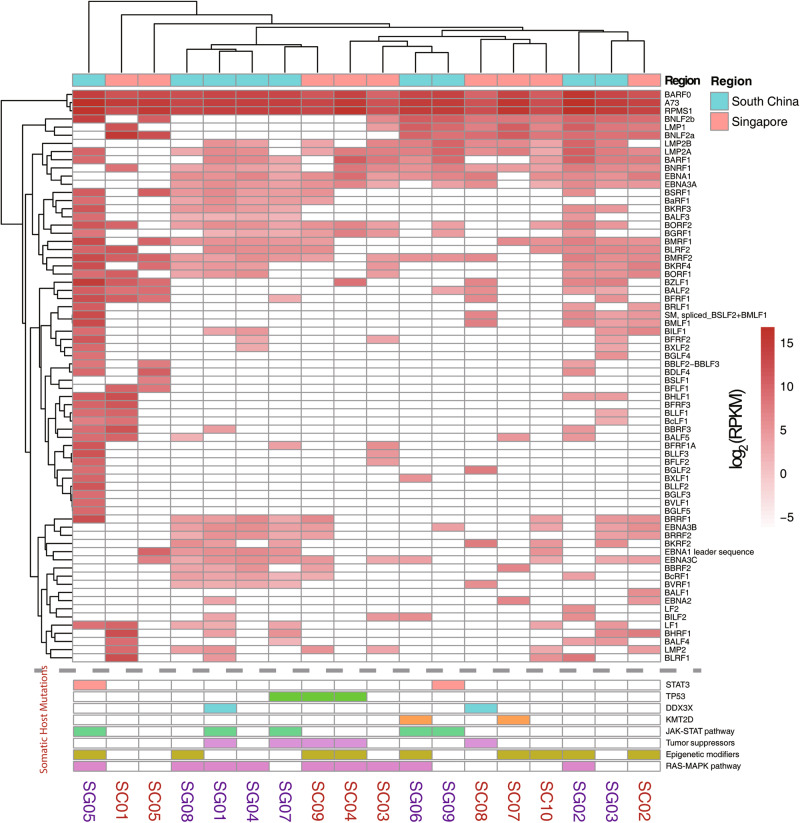
Expression profile of EBV genes in NKTCL. Expression at RNA transcription levels was normalized by using RPKM. Samples and genes were grouped according to their expression patterns based on hierarchical. Somatic host mutations in known driver genes and pathways in NKTCL are indicated in bottom heatmap

We further investigated the expression profile of EBV genes in 18 NKTCL samples, which transcriptome data were available. Even though transcripts were found covering almost all the regions of EBV genome, high coverages were restricted to regions at 6–7 kb and 138–160 kb (Fig. 1). Most of the latent genes were expressed in NKTCL, including those of *EBNA* family, *LMP* family and *BARTs* (Fig. 5). High expression of *BARF0*, *A73*, and *RPMS1* at BART region were observed (RPKM >100 in at least half of the samples). Moreover, the lytic genes including *BNLF2a* and *BNLF2b* were also highly expressed (RPKM >100 in at least half of the samples) in NKTCL tissues. We shortlisted EBV genes with transcripts observed in at least half of the samples and identified three gene-sets with correlated expression (Figure S11). Set-1 includes *BNRF1*, *BORF1*, *BLRF2*, *LMP1*, and *BNLF2a*; Set-2 includes *BGRF1*, *BORF2*, *BMRF2*, *BKRF4*, *BMRF1*, *BRRF1* and *BNLF2b*; and Set-3 includes *BARF1*, *LMP2A*, *A73*, *BARF0*, and *RPMS1*. Notably, genes in Set-3 shared common promoter region, which might be result of transcriptional coactivation at the gene cluster.

Using hierarchical clustering, we found a set of EBV genes were significantly over-expressed in the samples with long-fragment-deletion EBV (Figure S12). Moreover, we observed lower expression of *RPMS1* in the sample carrying large-fragment deletion at the upstream of *RPMS1* (SC01), as compared with the average expression of *RPMS1* in the NKTCL samples (Fig. 5); and we didn't found primary transcript of BART miRNAs in the sample with ~10 kb deletion in BARTs region (SC07; Figure S13). These indicated that expression heterogeneity could be partially explained by deletion or mutation of EBV genome. Noteworthily, in the sample with EBV integration into the inter-exonic region of *NHEJ1*, the expression of *NHEJ1* is lower (0.45-fold) than the other samples (Figure S14). We found similar expression pattern of EBV genes between NKTCL samples from Southern China and Singapore, and none of these genes showed significant difference in expression between these two groups (data not shown). In addition, no significant correlation was identified between EBV transcriptional profiles and somatic host mutations.

### Disease-specific EBV expression profile

The expression profile of EBV derived from NKTCL was compared with those of other cancers retrieved from the published transcriptome data [11, 33] (Figure S15). The transcriptions of a set of latent genes, including *EBNA1*, *LMP2*, *A73*, *BARF0*, and *RPMS1*, were significantly lower in NKTCL than in NPC or GC; and the lytic genes, including *BALF* family and *LF* family are also less transcribed in NKTCL, whereas lytic genes including *BBRF3*, *BLRF2*, and *BSRF1* were significantly over-expressed in NKTCL as compared to the other cancers.

### Variations in T-cell epitopes in NKTCL-derived EBV

**Fig. 6:**
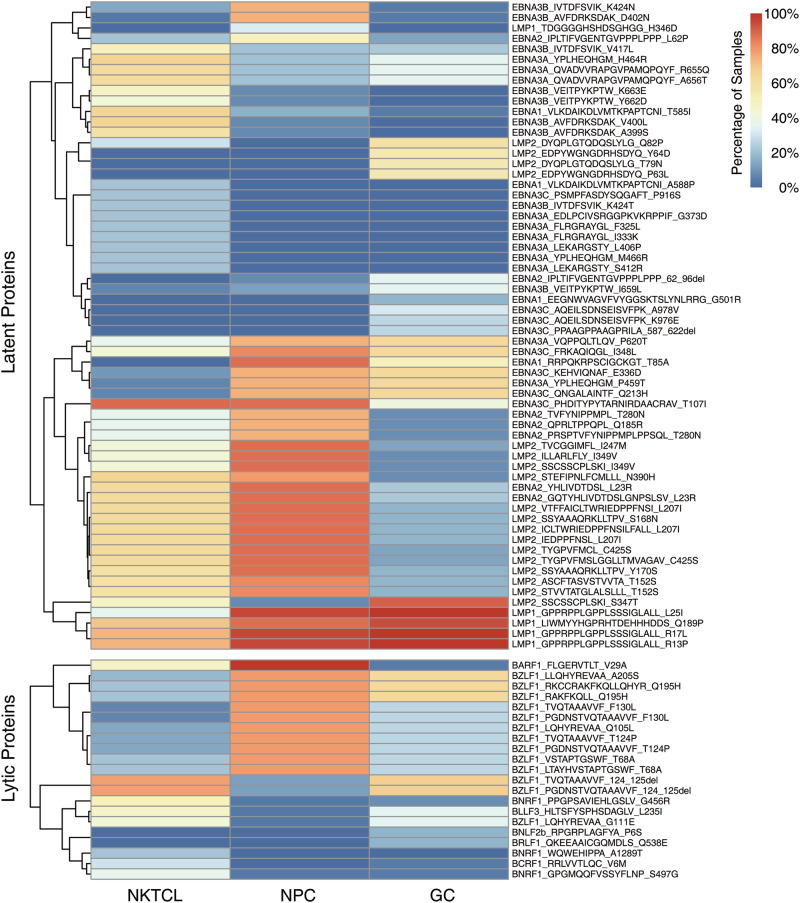
Frequencies for variations of T-cell epitope among NKTCL, NPC, and GC. Heatmaps show variations of T-cell epitope with frequencies significantly differ between NKTCL and NPC/GC (*p* < 0.05, Fisher’s exact test)

EBV encoded proteins might be the targets of immune recognition during its persistent infection, and their non-synonymous variations would alter recognition by immune cells [28]. Here, alterations of the known T-cell epitopes were examined in EBV sequences derived from NKTCL, NPC and GC, focusing on the EBV genes with at least one transcript in the NKTCL transcriptome (Table S3). A few hundred alterations of T-cell epitopes were detected in EBV derived from NKTCL (364), NPC (285) and GC (205) samples, respectively. Fifty-seven T-cell epitope mutations of EBV had significant difference in their frequencies between NKTCL and NPC, whereas there were 64 between NKTCL and GC (*p* < 0.05). Notably, 21 of these epitopes with significant enrichment in NKTCL samples were restricted to six EBV genes, including EBNA3A (G373D, F325L, I333K, L406P, S412R, H464R, M466R, T585I, and A588P), EBNA3B (A399S, V400L, V417L, K424T, Y662D, and K663E), EBNA3C (P916S), BARF1 (V29A), BCRF1 (V6M), and BNRF1 (G456R, S497G, and A1289T). Moreover, 62 of 83 (74.7%) epitopes with significant difference in the frequencies between NKTCL and either NPC or GC are transcripts of the latent EBV genes, including *EBNA* family, *LMP1* and *LMP2*; whereas, the remaining 21 epitopes are of lytic genes, including *BZLF1*, *BLLF1*, *BARF1*, *BCRF1*, *BNRF1*, *BNLF2b*, and *BRLF1* (Fig. 6).

## Discussion

NKTCL is one of the malignancies strongly associated with EBV. However, both of the genomic and the transcription profiles of EBV at genome-wide level have not yet been explored in NKTCL. For the first time, we assembled 27 EBV genomes directly from NKTCL clinical samples, and systematically characterized the mutational and transcriptional landscape of NKTCL-derived EBV.

We revealed multiple quasispecies of EBV in individual NKTCL samples, which were reflected by diverse mutations with enrichment in a few hotspots common in different diseases, such as *EBNA1*, *EBNA3*, and *LMP1* [8, 11], as well as a set of NKTCL-specific mutations/alterations in *BPLF1* and *EBNA* family (Figure S1). Phylogenetic tree analysis showed that EBV isolates derived from NKTCL samples tend to cluster closely, apart from clusters by other diseases. Moreover, as compared with other types of cancers, transcriptome analysis revealed lower transcription of both latent and lytic genes in NKTCL; and larger amount of T-cell epitope alterations was observed in NKTCL, suggesting that distinct mechanism of immune evasion might be deployed in NKTCL. These results indicate that EBV in NKTCL exhibits unique features in not only its sequence but also the expression signatures, supporting the hypothesis of the existence of disease-specific EBV. However, whether the unique EBV has been driving the development of NKTCL or simply adapted to the niche of NKTCL as bystander await further investigations.

After EBV infection, how the viral genome persistently maintains has been puzzled. First, whether the genomes propagate as episomal form or fully integrated into host genome or both is still debatable [34–36]. In our study, we managed to assemble large scaffolds and full-length EBV genome, but there is no sign of further tiling extension to human sequences, suggesting that EBV genome might be maintained as episomal form in NKTCL tumor cells, which is consistent with the observation in NPC or GC [37]. Second, impaired EBV genome with long-fragment deletion events were found prevalent in NKTCL samples, particularly for the region at 138–160 kb of EBV containing *BARTs*, *RPMS1, LF2*, and *LF3* with essential roles. BART miRNAs are well known oncogenic players in multiple cancers [38]; *RPMS1* has been reported as an oncogene in NPC [13, 39]; and LF2 could retard lytic replication of latent EBV by altering Rta subcellular localization [40, 41]. Considering that the region is highly accessible by transcription factors as revealed by the transcriptome analysis, disruption of the EBV encoded genes as a consequence of the deletion might contribute to NKTCL tumorigenesis. We note that large-deletion event is rarely reported by far in asymptomatic samples, we suspect that the clonal heterogeneity of EBV might reflect the unique features of tumor environment among NKTCL samples, where the germline background (such as HLA) of the host is also a contributor. Third, multiple EBV clones with both intact full-length and impaired or deleted EBV genomes were observed simultaneously in individual NKTCL samples. Since EBV infected solely the tumor cells as *EBERs* have been detected only in tumor cells in biopsy sections, we speculate that different EBV clones were present in different tumor cells of the specific individuals. This highlights the heterogeneity of tumor cells, which is not only reflected by the host alterations, but also the EBV genomes. As the deleted region harbors genes important for EBV latency and cell lysis, the subclone of tumor cells with impaired EBV genome might be endowed cell properties different from those with intact EBV genome.

Intriguingly, we also observed integrations of short EBV fragments into human chromosomes, coincident with episomal EBV genomes in NKTCL. Particularly, the EBV-host integration at *NHEJ1* likely results in the disruption of *NHEJ1* transcription. *NHEJ1* encodes a non-homologous end-joining factor, and it has been demonstrated that disruption of *NHEJ1* potentially leads to disordered DNA repairing and unstable host genome, and participates in tumorigenesis [42, 43]. Consistently, we observed higher number of structural variations in the sample with *NHEJ1* disruption (SG05; Figure S16). Other EBV-host integrations were found in repeat regions, including SINE and LINE, with a similar scenario as described in a previous study on hepatitis B virus (HBV) in liver cancer [32]. Integration of HBV to LINE1 results in a chimeric HBV-LINE1 transcript, which functions as a hybrid RNA and promotes liver cancer development through Wnt/β-catenin signaling pathway activation [32]. Moreover, it has been well documented that HPV integrations into tumor suppressors contribute to malignant transformation of host cells [44–48]. Therefore, integration of EBV short sequence into the host genome and the consequent disruption of the important host genes for maintaining the biological hemostasis might represent a novel carcinogenic mechanism of EBV, as similar scenarios for HPV and HBV [32, 44, 49, 50].

In summary, we have profiled the genomic and transcriptomic landscapes of EBV genome in NKTCL, and pointed out somatic events including disruptions of both EBV genome by large-fragment deletions and host genes by integration of short viral sequences. Our findings provide insights into the understanding of EBV’s role the etiology of NKTCL, as well as potential T-cell targets for immunotherapy against NKTCL. We acknowledge that further characterizations of the molecular events would provide more information on the exact mechanisms underlying their pathogenic potentials and clinical significance.

## Supplementary information

Figure S1. Mutation rates of EBV genomes originated from NKTCL, NPC and GC

Figure S2. Comparison of EBV mutation profiles between samples from Southern China and Singapore

Figure S3. Somatic mutation profiles of NKTCL genome

Figure S4. Different amino acid substitution between NKTCL samples with and without mutated JAK-STAT pathway

Figure S5. Deletion profile of EBV genome in NKTCL samples

Figure S6. Schematic presentation for multiple EBV clones in individual NKTCL samples

Figure S7. Cross-platform validation of EBV-host integration site at human *NHEJ1* gene

Figure S8. Numbers of EBV variants among different cancers

Figure S9. Comparison of amino acid substitutions between NKTCL-derived and NPC-derived EBV

Figure S10. Comparison of amino acid substitutions between NKTCL-derived and GC-derived EBV

Figure S11. Correlations in expression of EBV genes in NKTCL samples

Figure S12. EBV genes significantly over-expressed in samples with long-fragment-deletion EBV

Figure S13. Transcriptional coverage in NKTCL-derived EBV with genomic deletion

Figure S14. Expression level of *NHEJ1* in the *NHEJ1*-disrupted NKTCL sample (SG05) as compared with the other NKTCL samples

Figure S15. Differentially expressed EBV genes between NKTCL and other cancers

Figure S16. Number of structural variations in the *NHEJ1*-disrupted sample (SG05) as compared with the other NKTCL samples

Table S1. Clinical information of NKTCL patients

Table S2. Accession numbers of NKTCL-derived EBV genomes

Table S3. Alterations of known T-cell epitopes in NKTCL
