# Supplementary figures and images for: Genomic and transcriptomic landscapes of Epstein-Barr virus in extranodal natural killer T-cell lymphoma
Source: Leukemia. 2018 Dec 13;33(6):1451–62. doi: 10.1038/s41375-018-0324-5 (PMC6756073; doi:10.1038/s41375-018-0324-5)

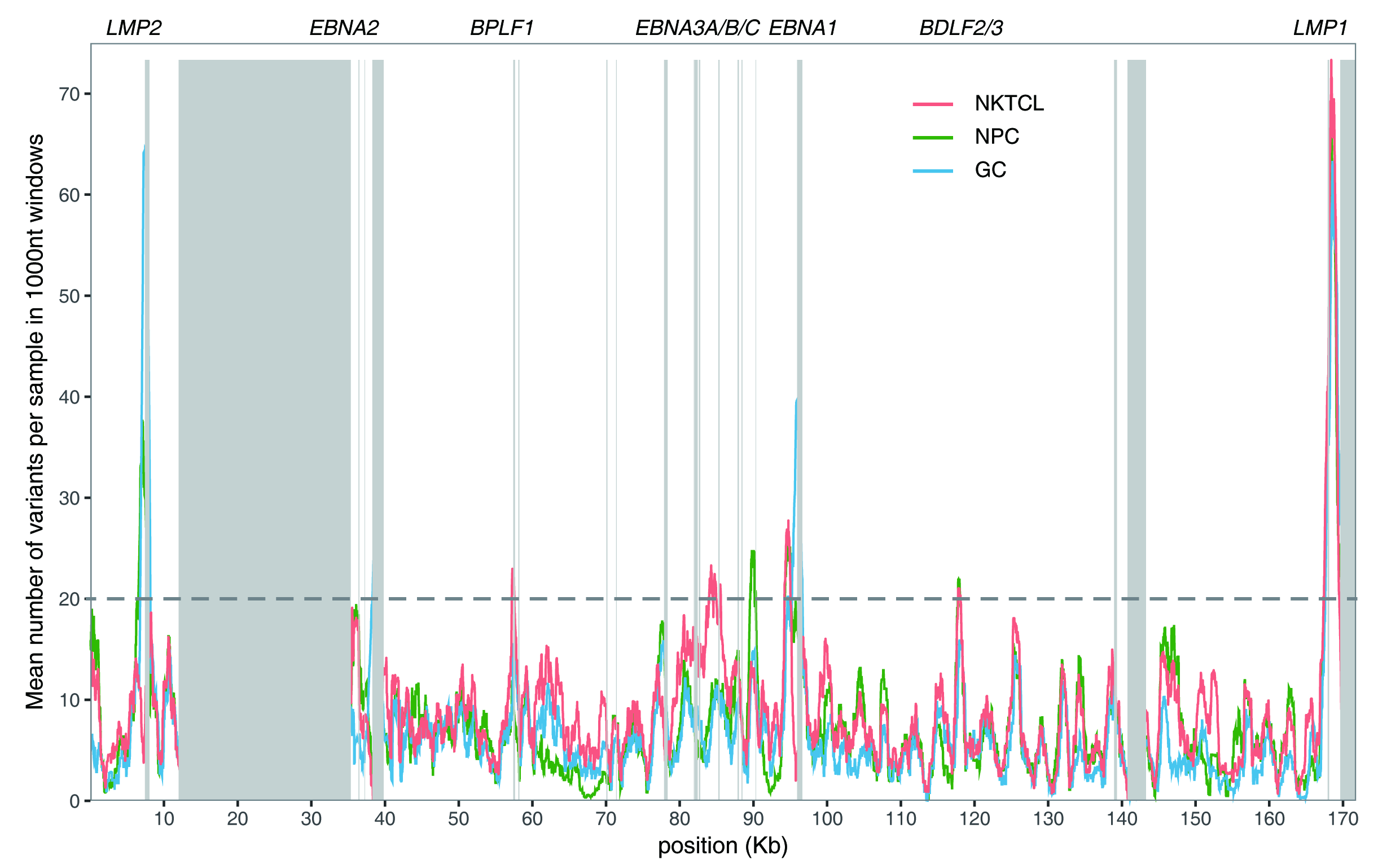

Supplement: Supplementary file 1 — Figure S1. Mutation rates of EBV genomes originated from NKTCL, NPC and GC [file 41375_2018_324_MOESM1_ESM.tif]

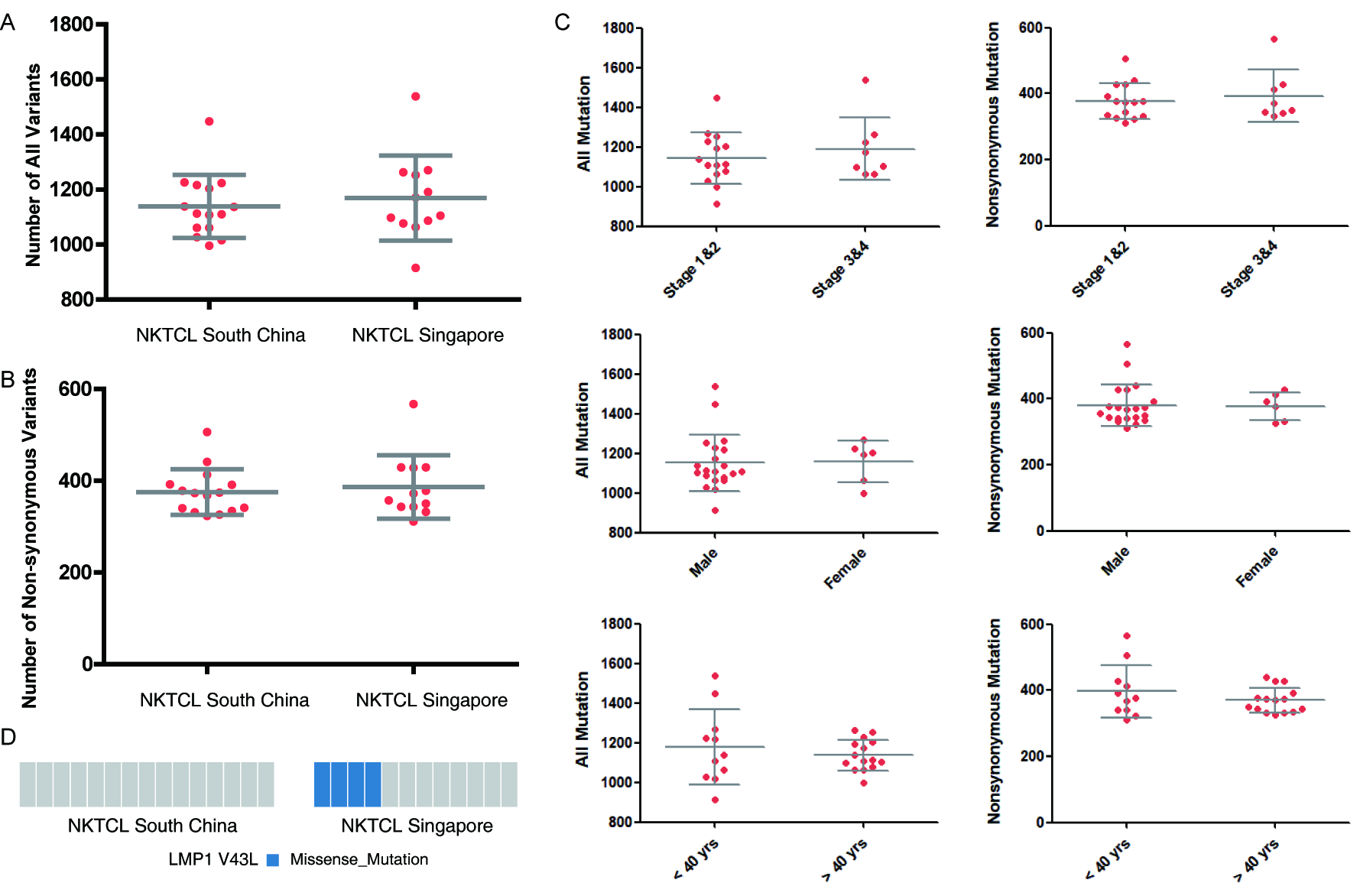

Supplement: Supplementary file 2 — Figure S2. Comparison of EBV mutation profiles between samples from Southern China and Singapore [file 41375_2018_324_MOESM2_ESM.tif]

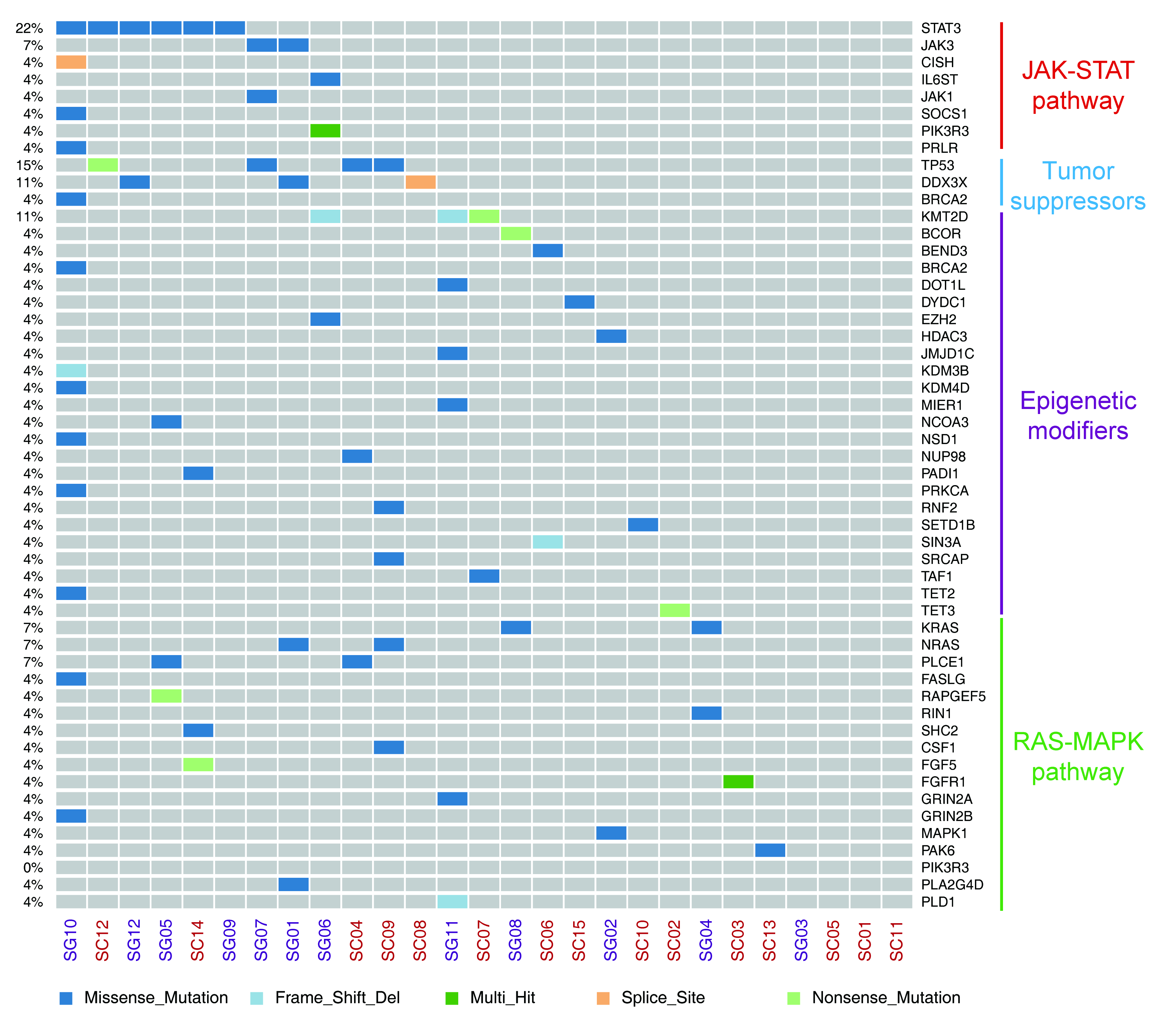

Supplement: Supplementary file 3 — Figure S3. Somatic mutation profiles of NKTCL genome [file 41375_2018_324_MOESM3_ESM.tif]

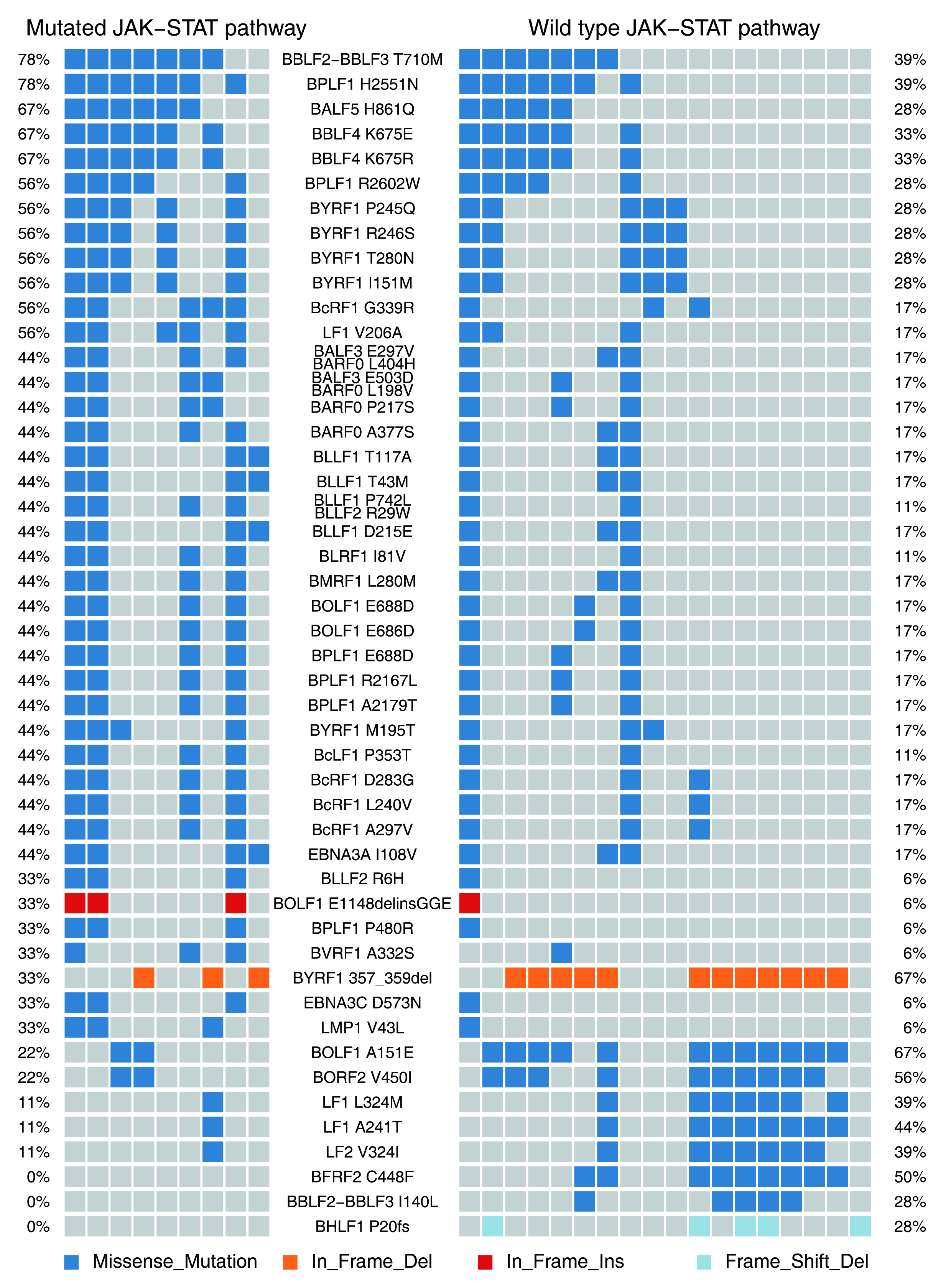

Supplement: Supplementary file 4 — Figure S4. Different amino acid substitution between NKTCL samples with and without mutated JAK-STAT pathway [file 41375_2018_324_MOESM4_ESM.tif]

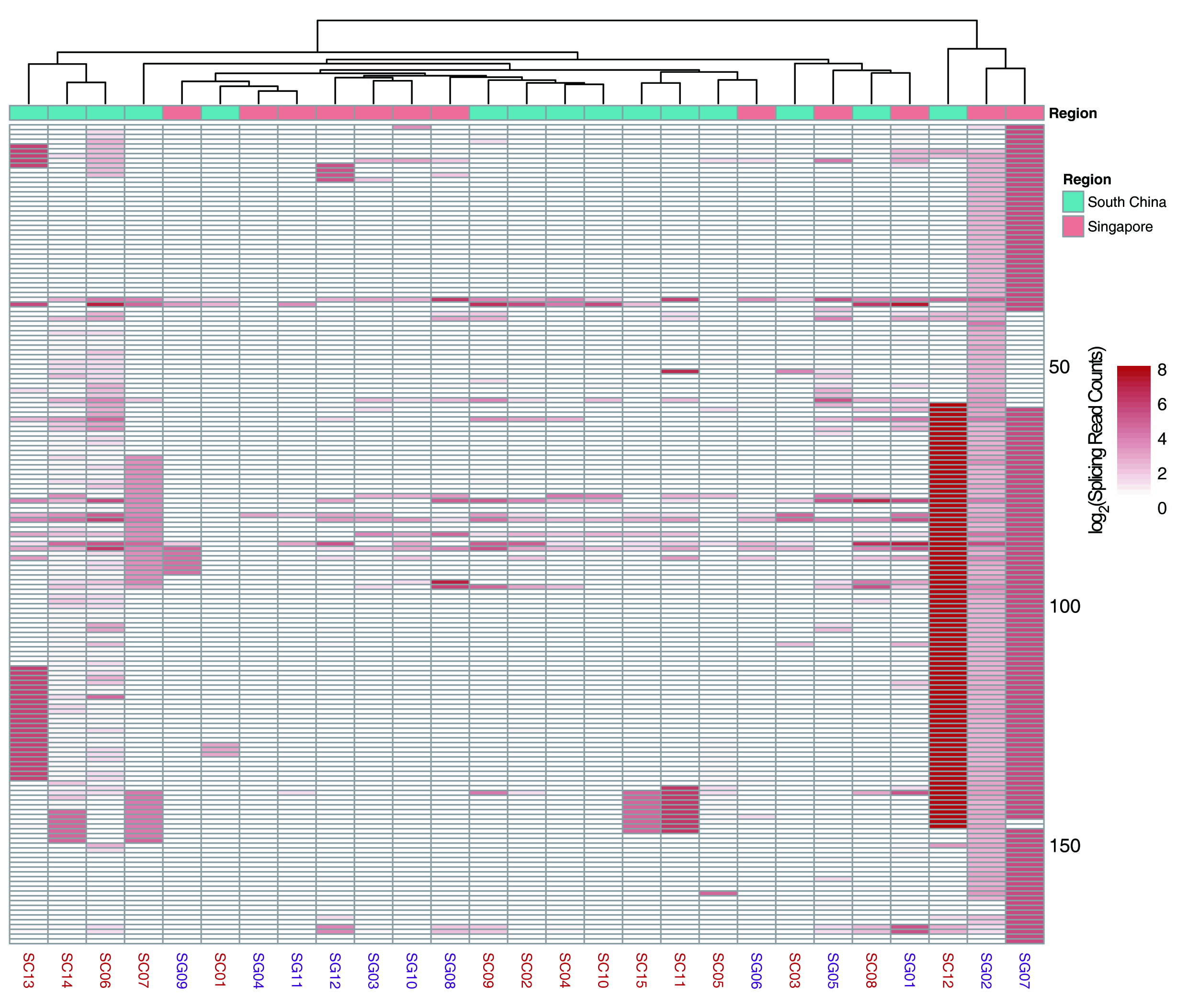

Supplement: Supplementary file 5 — Figure S5. Deletion profile of EBV genome in NKTCL samples [file 41375_2018_324_MOESM5_ESM.tif]

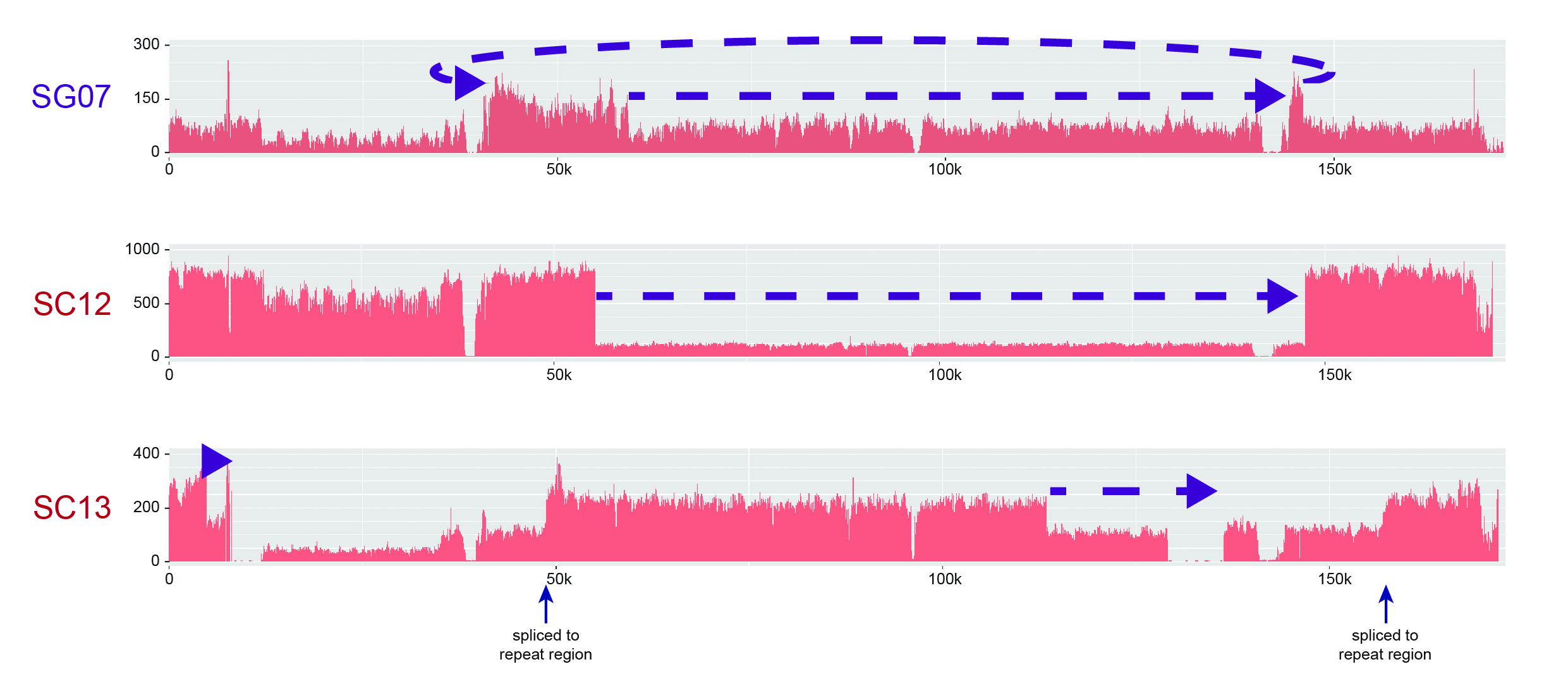

Supplement: Supplementary file 6 — Figure S6. Schematic presentation for multiple EBV clones in individual NKTCL samples [file 41375_2018_324_MOESM6_ESM.tif]

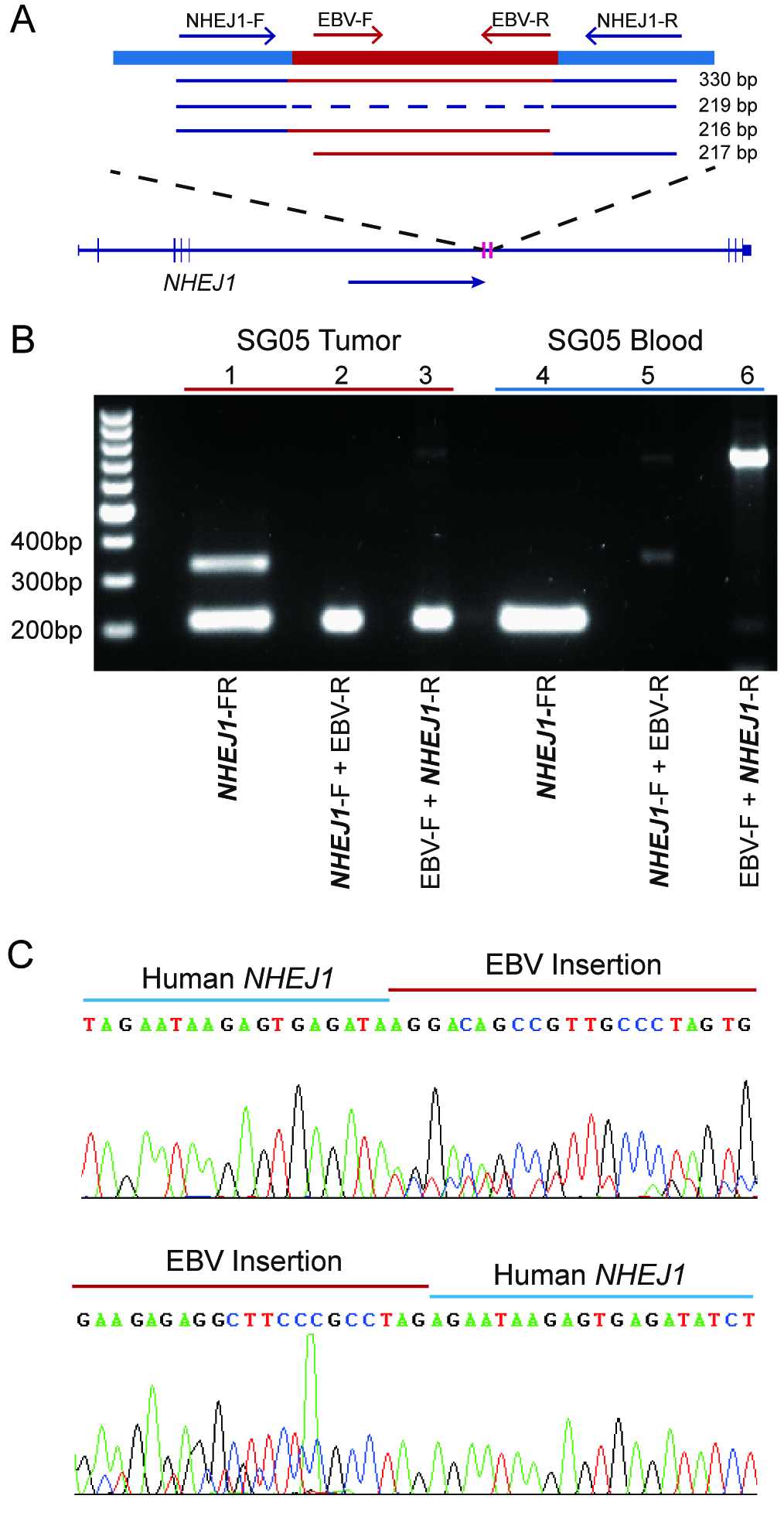

Supplement: Supplementary file 7 — Figure S7. Cross-platform validation of EBV-host integration site at human NHEJ1 gene [file 41375_2018_324_MOESM7_ESM.tif]

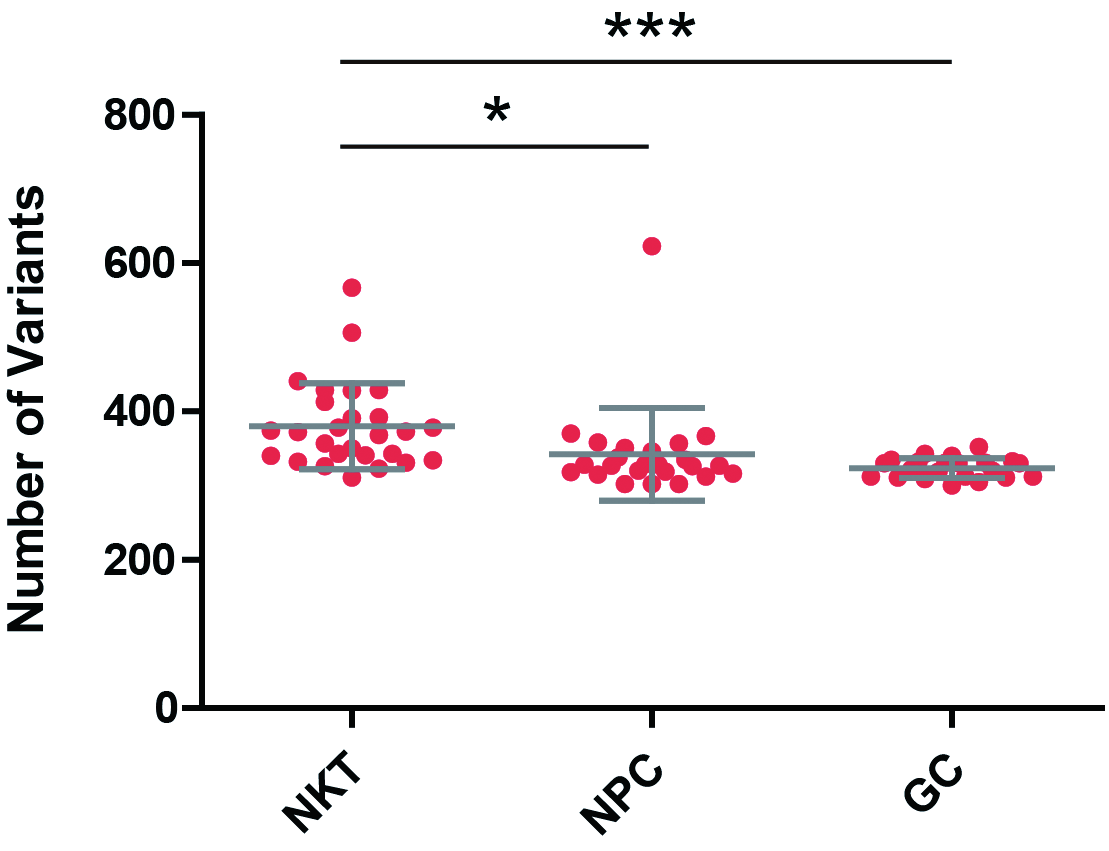

Supplement: Supplementary file 8 — Figure S8. Numbers of EBV variants among different cancers [file 41375_2018_324_MOESM8_ESM.tif]

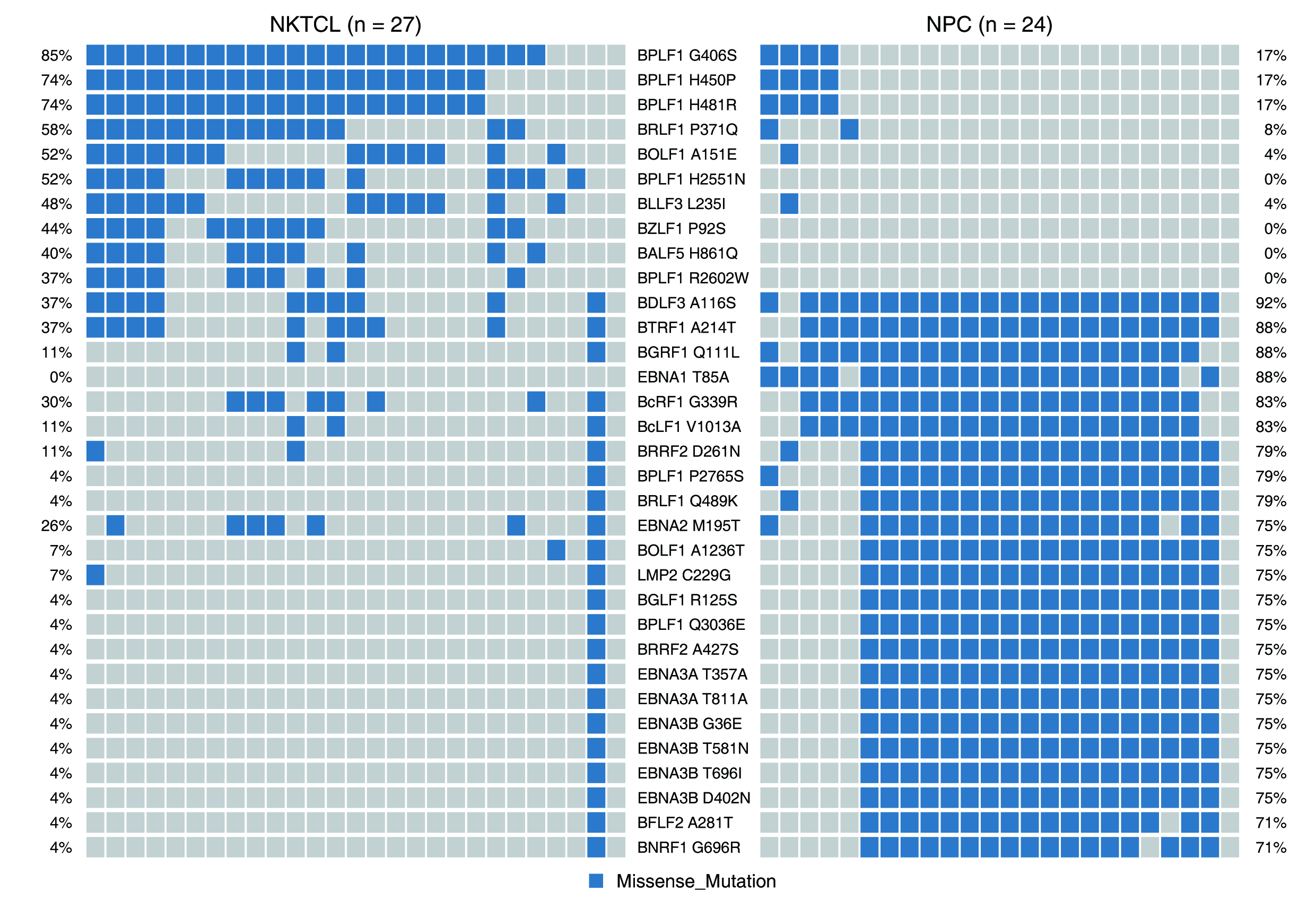

Supplement: Supplementary file 9 — Figure S9. Comparison of amino acid substitutions between NKTCL-derived and NPC-derived EBV [file 41375_2018_324_MOESM9_ESM.tif]

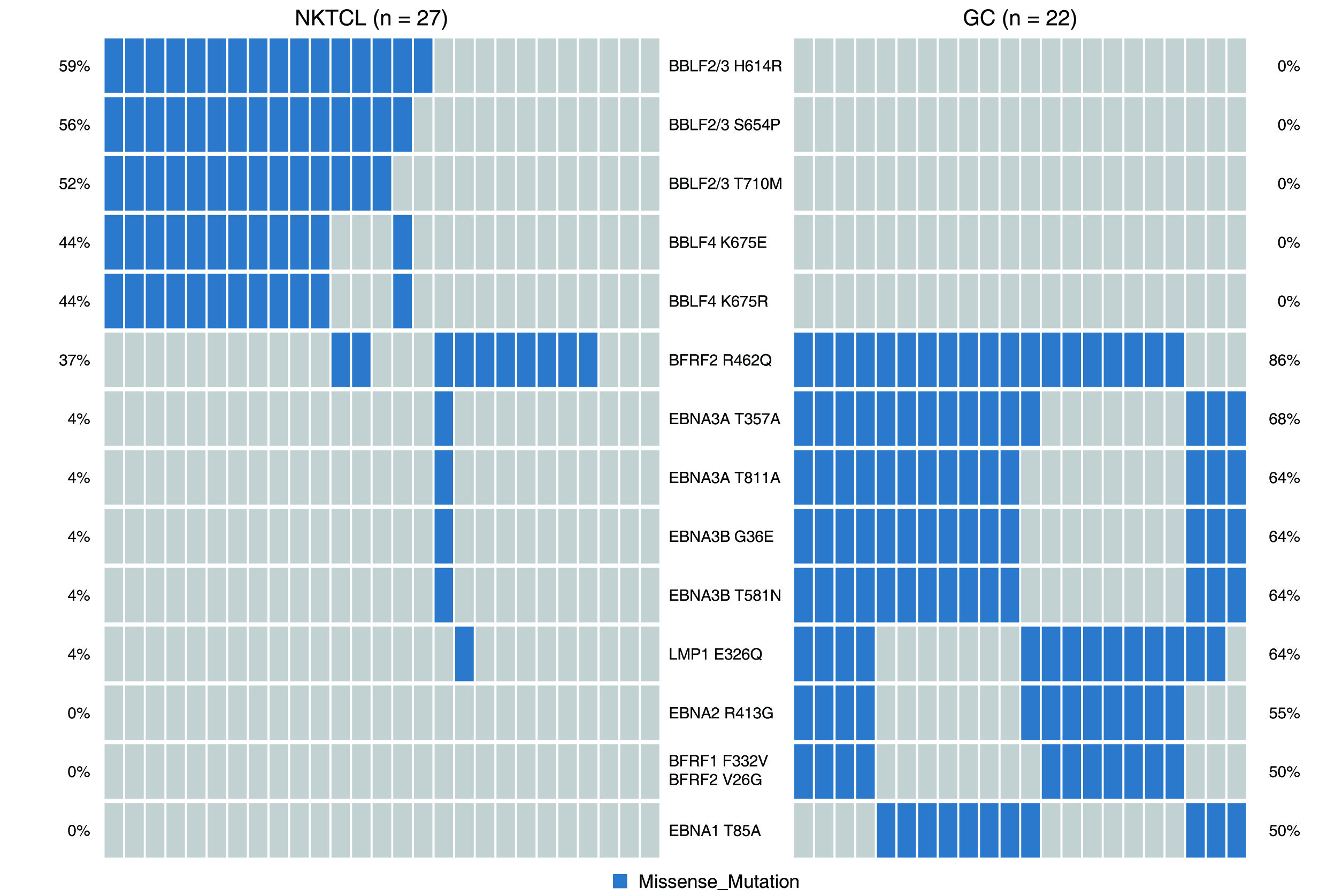

Supplement: Supplementary file 10 — Figure S10. Comparison of amino acid substitutions between NKTCL-derived and GC-derived EBV [file 41375_2018_324_MOESM10_ESM.tif]

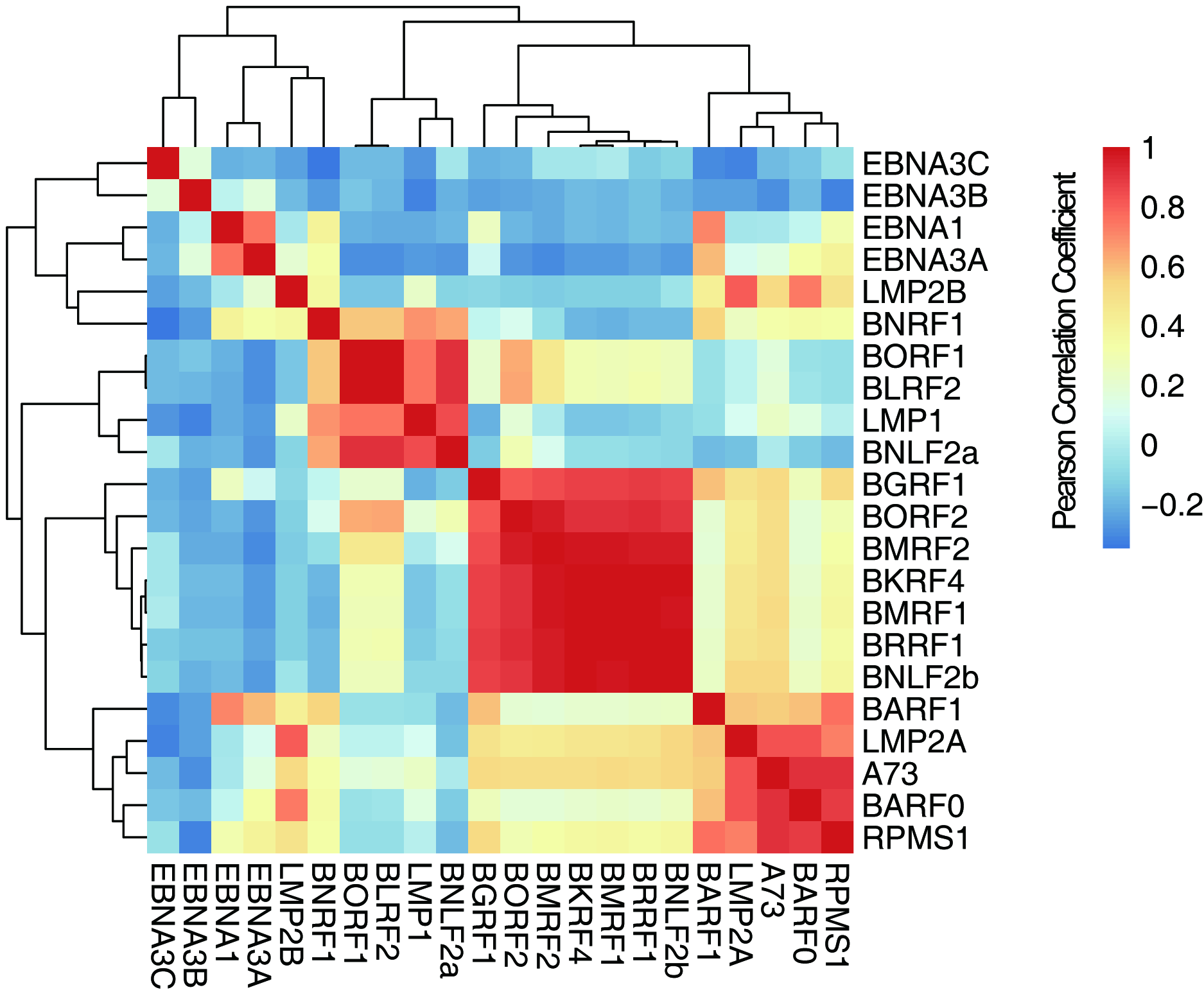

Supplement: Supplementary file 11 — Figure S11. Correlations in expression of EBV genes in NKTCL samples [file 41375_2018_324_MOESM11_ESM.tif]

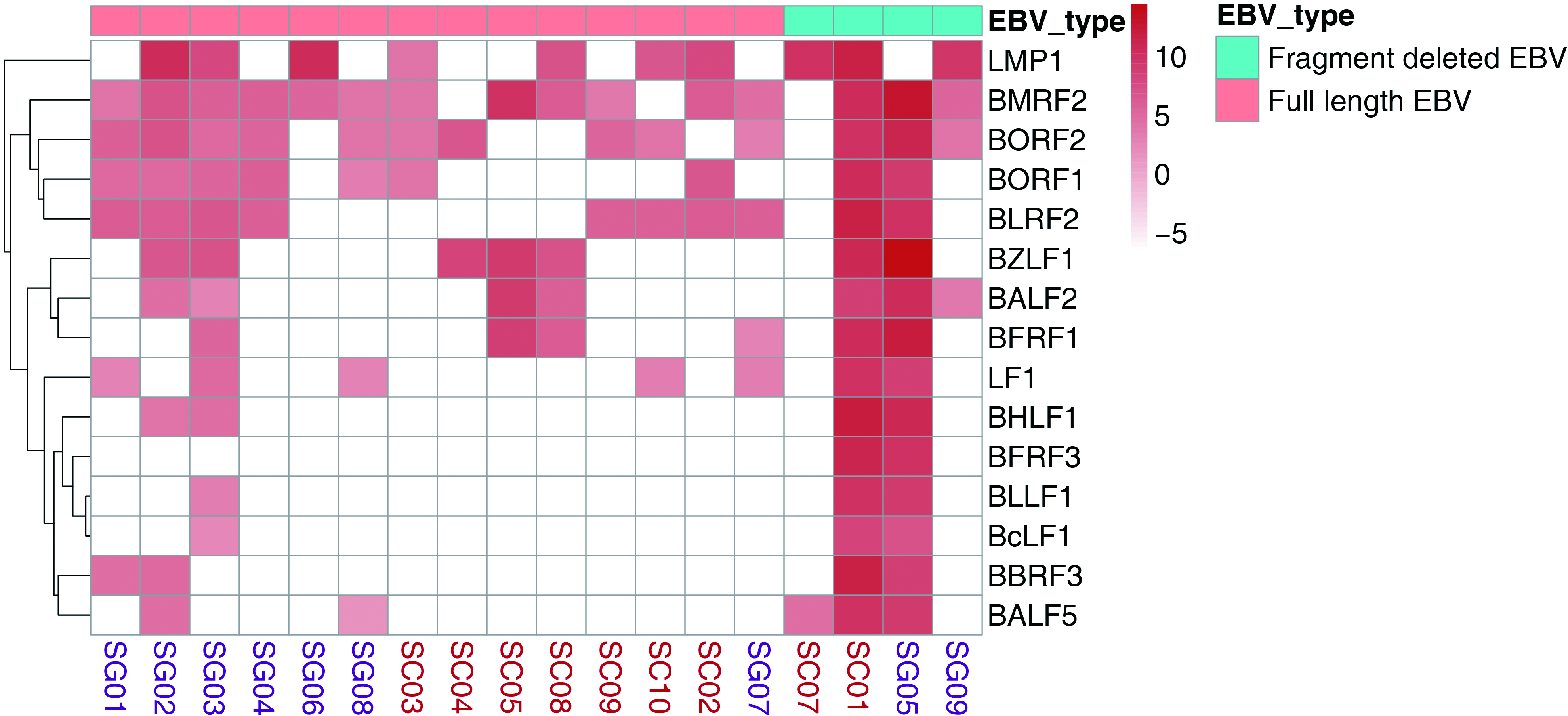

Supplement: Supplementary file 12 — Figure S12. EBV genes significantly over-expressed in samples with long-fragment-deletion EBV [file 41375_2018_324_MOESM12_ESM.tif]

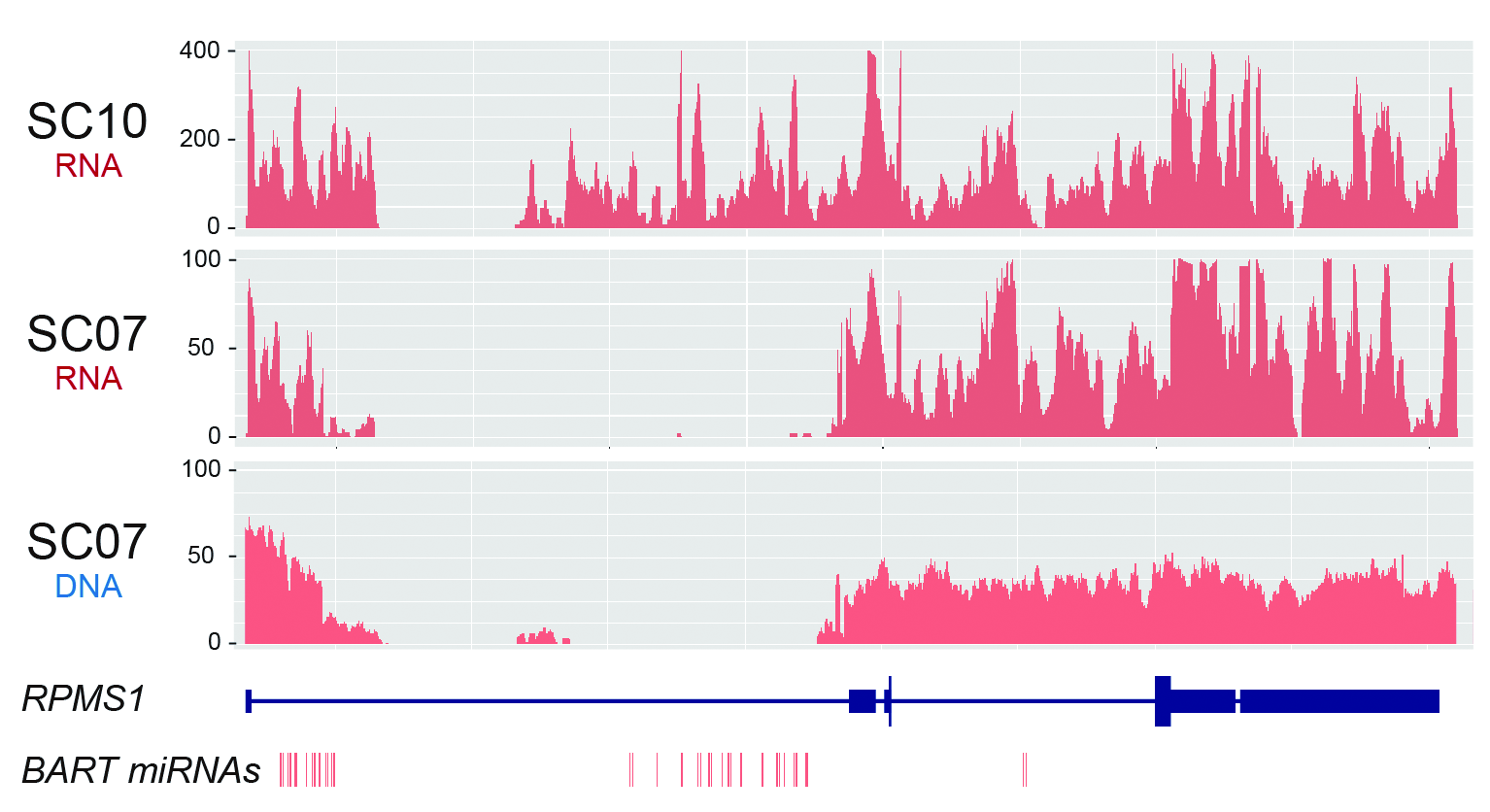

Supplement: Supplementary file 13 — Figure S13. Transcriptional coverage in NKTCL-derived EBV with genomic deletion [file 41375_2018_324_MOESM13_ESM.tif]

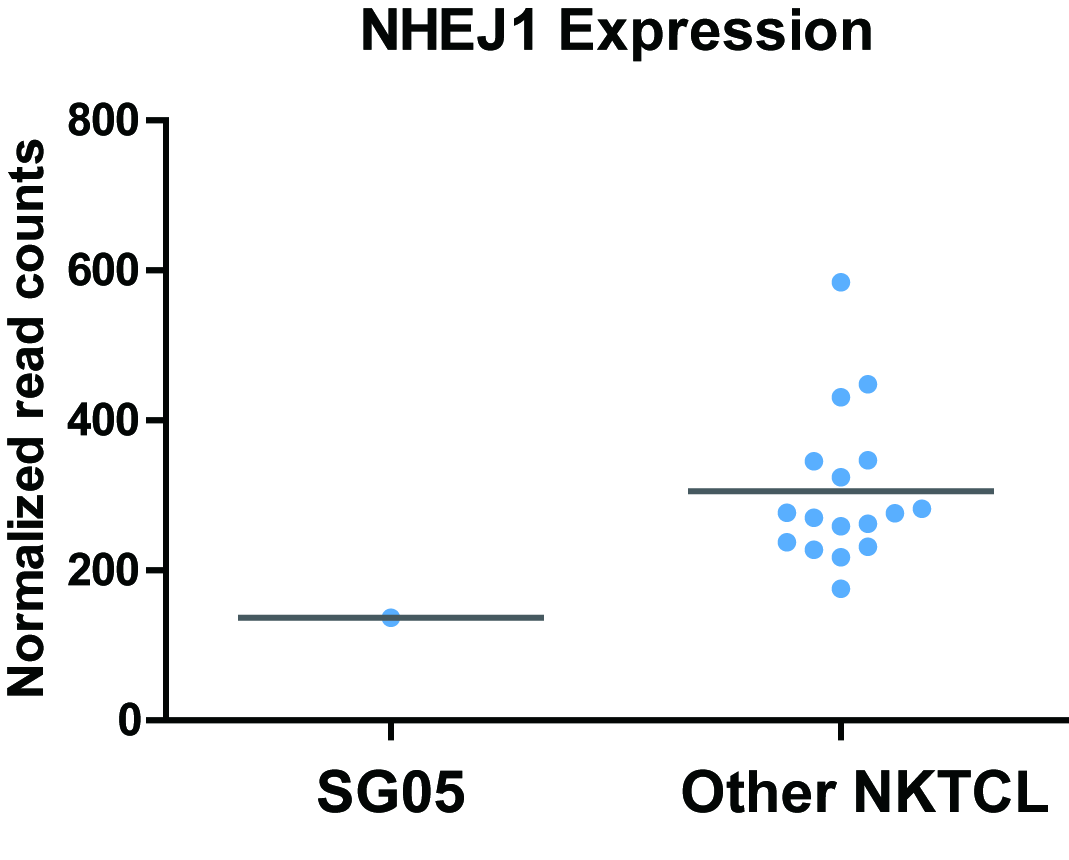

Supplement: Supplementary file 14 — Figure S14. Expression level of NHEJ1 in the NHEJ1-disrupted NKTCL sample (SG05) as compared with the other NKTCL samples [file 41375_2018_324_MOESM14_ESM.tif]

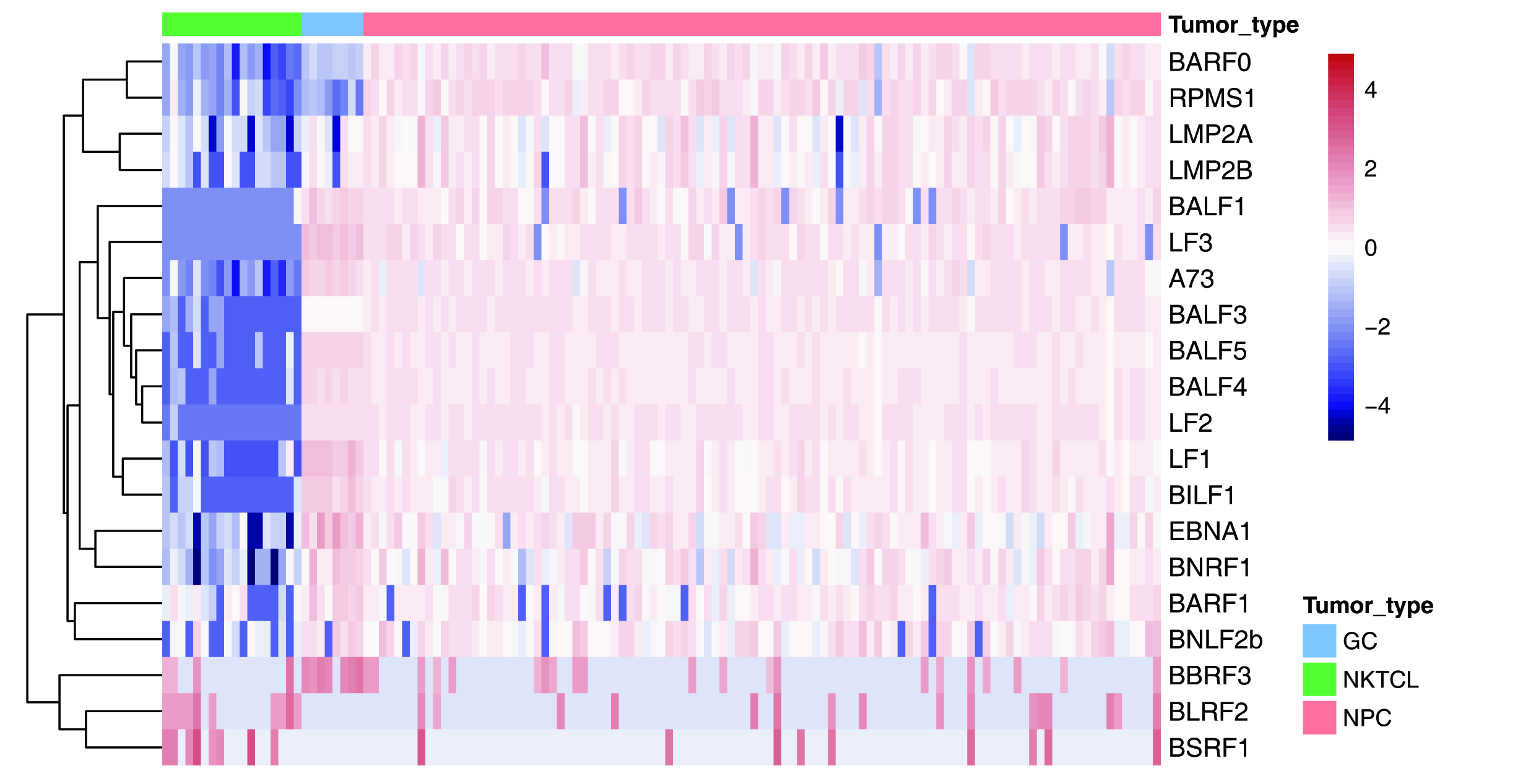

Supplement: Supplementary file 15 — Figure S15. Differentially expressed EBV genes between NKTCL and other cancers [file 41375_2018_324_MOESM15_ESM.tif]

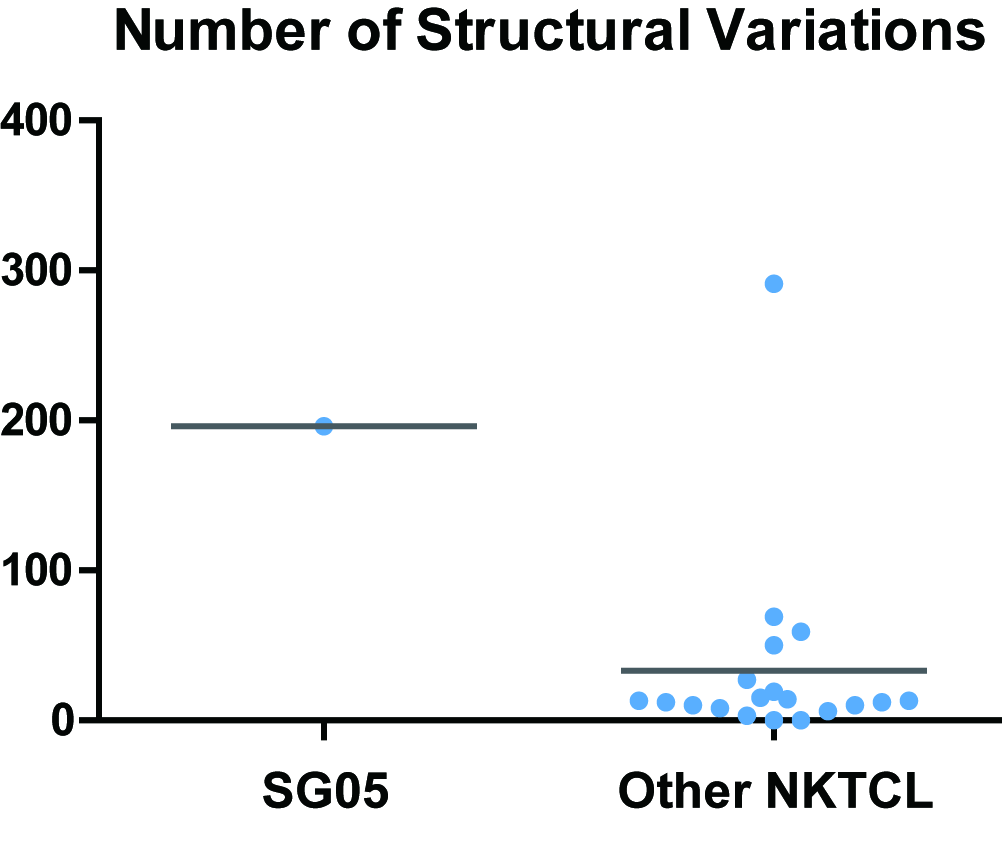

Supplement: Supplementary file 16 — Figure S16. Number of structural variations in the NHEJ1-disrupted sample (SG05) as compared with the other NKTCL samples [file 41375_2018_324_MOESM16_ESM.tif]
